# Pharmacologic inhibition of MEK1/2 reduces lung inflammation without impairing bacterial clearance in experimental *Pseudomonas aeruginosa* pneumonia

**DOI:** 10.1186/s41479-017-0037-y

**Published:** 2017-09-05

**Authors:** Matthew E. Long, Ke-Qin Gong, William E. Eddy, W. Conrad Liles, Anne M. Manicone

**Affiliations:** Center for Lung Biology, Division of Pulmonary, Critical Care and Sleep Medicine, University of Washington, 850 Republican St, Seattle, WA 98109 USA

**Keywords:** Macrophage, MEK1/2, *Pseudomonas aeruginosa*, Lung

## Abstract

This study was designed to test the therapeutic potential of a MEK1/2 inhibitor (MEKi) in an experimental model of *Pseudomonas aeruginosa* pneumonia. The study found that treatment with MEKi reduced alveolar neutrophilic inflammation and led to faster recovery of weight compared to carrier-treated mice, without impairing bacterial clearance. Alveolar macrophages isolated from MEKi-treated mice also had increased M2 gene and protein expression, supporting the concept that MEKi modulates in vivo macrophage inflammatory responses. In summary, this report demonstrates the potential of MEKi to promote the resolution of inflammation in vivo during a primary lung infection without impairing bacterial clearance.

## Introduction

*Pseudomonas aeruginosa* pulmonary infections are common in hospitalized patients and are one of the drivers of progressive lung decline in patients with cystic fibrosis [1]. Recurrent or chronic *P. aeruginosa* infections result in increased lung inflammation, subjecting the lung to host-mediated tissue damage. Immune modulating strategies to reduce deleterious lung inflammation without compromising host-defense mechanisms may be therapeutically beneficial in this situation, and new strategies to reduce lung injury are needed.

The mitogen-activated protein kinases MEK1 (*Map2k1*) and MEK2 (*Map2k2*) participate in intracellular signaling networks and exert control on the downstream effector molecules ERK1 and ERK2, via MEK1/2-dependent serine and tyrosine phosphorylation [2]. Several recent studies, including this one, have investigated the potential therapeutic use of MEKi compounds as immune modulating agents [3–9]. Given that a MEKi is now FDA-approved for use in certain cancer chemotherapeutic regimens [10], the potential for application of these pharmaceuticals to fulfill unmet clinical needs in infectious diseases is intriguing.

We recently reported [3] that MEKi delivery to mice, starting 1 day post- lipopolysaccharide (LPS) acute lung injury (ALI), reduced lung neutrophilia and led to faster resolution of lung injury and greater macrophage M2 polarization. These findings suggest that MEK pathways may be important therapeutic targets; however, information on the therapeutic use of MEKi in bacterial pneumonia models is lacking. The goals of the current study were to determine if MEKi promotes the resolution of inflammation in vivo during experimental *P. aeruginosa* pneumonia in mice without increasing susceptibility to infection or compromising bacterial clearance.

## Methods

### Animal ethics

This study used age (8–12 weeks) and sex-matched C57BL/6J mice that included both male and female groups. The University of Washington Institutional Animal Care and Use Committee reviewed and approved all animal procedures prior to initiation of these studies.

### Bacterial strain and culture conditions

*P. aeruginosa* strain K (PAK) was grown overnight in lysogeny broth (LB-Miller) at 37°C. Bacteria were pelleted at 13,000 x *g*, washed twice in phosphate buffer saline (PBS), and re-suspended in PBS so that a target inoculum of 5 × 10^6^ colony-forming units (CFU) in 50 μl was delivered via oropharyngeal aspiration.

### Infection, drug treatment, and sample processing

C57BL/6J mice were anesthetized using isoflurane and positioned for oropharyngeal PAK infection. After instillation, mice were monitored daily for weight change and activity. MEKi PD0325901 (20 mg/kg, InvivoGen, San Diego, California [CA], United States of America [USA]) or carrier control (10% dimethylsulfoxide [DMSO] in PBS) was delivered by intraperitoneal (IP) injection on days 2 and 3 after infection. Animals were sacrificed on day 4 after infection. Alveolar immune cells were collected by three serial bronchoalveolar lavage (BAL) with PBS + 5 mM EDTA. BAL cells were centrifuged for 10 min at 350 x *g* and re-suspended in sterile PBS. Total BAL cells were enumerated on a Cellometer Auto 2000 (Nexcelom Bioscience, Lawrence, Massachusetts [MA], USA) and 50,000 cells were used for slide cytospin preparations and stained using Diff-Quik (Siemens, Newark, Delaware, USA). Random fields of view from slides at a magnification of 40× were observed to determine the percentage of neutrophils and mononuclear cells in each sample. A minimum of 200 total cells from each slide was counted.

### Quantitative real-time polymerase chain reaction

The remaining cells from day 4 BAL were plated in 24-well tissue culture plates and incubated for 60 min at 37 °C, 5% CO_2_, to allow for alveolar macrophage adherence. Wells were rinsed 3 times with PBS to remove non-adherent cells and total macrophage ribonucleic acid (RNA) was isolated with a NucleoSpin RNA isolation kit (Clontech Laboratories, Mountain View, CA, USA). A cDNA template was created from RNA by the High-Capacity cDNA Reverse Transcription kit (Applied Biosystems, Foster City, CA, USA) that was then used in quantitative real-time PCR (qPCR) reactions with validated TaqMan FAM-labeled primer probes for *Hprt*, *Retnla*, *Ccl17*, *Arg1*, and *Tgfb1* (Life Technologies, Carlsbad, CA, USA) using the SensiMix II Probe Hi-ROX Kit (Bioline, Taunton, MA, USA). Reactions were performed on an ABI 7900HT Fast Real-Time PCR System. Duplicate replicates from each sample were used to obtain the average cycle threshold (Ct) and the ΔCt for each primer probe relative to *Hprt* was calculated as previously described [3]. Samples were normalized to the average of carrier-treated mice from an individual experiment.

### BAL fluid total protein, immunoglobulin M, and CXCL1/KC measurements

Cell free BAL fluid (BALF) was obtained from mice at day 4 post-infection and stored at −80 °C. Aliquots were used to measure total protein by a bicinchoninic acid (BCA) assay (Pierce, Rockford, Illinois, USA), IgM (Bethyl Laboratories, Inc., Montgomery, Texas, USA), and CXCL1/KC (R&D Systems, Minneapolis, Minnesota, USA) by enzyme-linked immunosorbent assay (ELISA). Standard manufacturer’s protocols were followed for each assay.

### Flow cytometry

Multicolor flow cytometry on lung homogenates prepared from the right lobe were used to measure surface expression of transferrin receptor (CD71) on alveolar macrophages. Alveolar macrophages were identified as CD45^+^/Ly6G^−^/CD11c^+^/SigF^+^ cells. The median fluorescence intensity (MFI) for CD71-PerCP/Cy5.5 was determined using Flowjo software (TreeStar, Ashland, Oregon, USA) The ΔMFI was calculated by subtracting the MFI of isotype-stained from the MFI of CD71-stained samples, as previously described [3, 11]. A Canto RUO (BD Biosciences) was used for acquisition of data.

### Quantification of bacterial burden

After sacrifice and lavage, the left lung and the spleen were removed and each tissue sample placed in 1 ml of sterile PBS. A sterile sonicator tip was immersed into the PBS and tissues were homogenized by sonication for 30 s. Tissue homogenates were subjected to serial dilution and spots were plated on *Pseudomonas* isolation agar (Sigma-Aldrich, St. Louis, Missouri, USA). Plates were incubated at 37 °C for 1–2 days and colonies were enumerated when visible. Organs from uninfected mice and from aliquots of sterile PBS used in making dilutions were plated to confirm that procedures were performed under sterile conditions.

### Statistics

Statistical analyses were performed using GraphPad Prism 6 software. Normal Gaussian distribution was assessed by the D’Agostino–Pearson normality test using an alpha = 0.05. Samples in which Gaussian distribution were assumed were analyzed by the unpaired *t* test to compare groups, while samples in which a Gaussian distribution was not assumed were analyzed using the nonparametric Mann-Whitney *t* test. Two-way analysis of variance (ANOVA) with Bonferroni’s multiple comparisons was used where appropriate. Statistical significance was considered as *p* < 0.05.

## Results

### MEKi reduces inflammation without inhibiting bacterial clearance during experimental *Pseudomonas aeruginosa* pneumonia

**Fig. 1:**
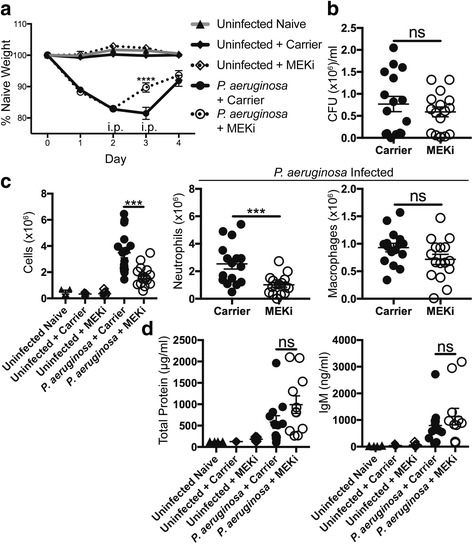
MEKi delivered after *P. aeruginosa* infection reduces inflammation without impairing bacterial clearance. C57BL/6J mice were infected with a target inoculum of 5 × 10^6^ CFU of *P. aeruginosa* by oropharyngeal instillation. **a** Animal weight was monitored over 4 days and IP injections of PBS + MEKi or PBS + carrier control were given to groups on days 2 and 3 post-infection. Mice receiving MEKi had significantly reduced weight loss on day 3 compared to carrier-treated animals, while treatments of uninfected mice did not alter animal weight. Data shown are mean ± SEM of 7 mice for each *P. aeruginosa* infected group from one representative experiment of three; the weights from additional controls of uninfected naïve (*n* = 4), uninfected carrier-treated (*n* = 7), and uninfected MEKi-treated (*n* = 7) are also included in this graph. Two-way ANOVA with Bonferroni’s multiple comparisons was used to analyze results. **b** Animals were euthanized 4 days after infection and lungs were homogenized in sterile PBS and plated for colony-forming unit (CFU) enumeration. There was no statistical difference in lung CFU between *P. aeruginosa* infected carrier and MEKi-treated groups (*n* = 16/group), demonstrating that bacterial clearance was not impaired by MEKi-treatment. **c** Total BAL cells, neutrophils and macrophages were enumerated and identified on Diff-quick cytospin preparation (*n* = 16/ *P. aeruginosa* infected group) and analyzed by parametric unpaired *t*-test. **d** Cell-free BALF was used to measure total protein and IgM (carrier *n* = 12, MEKi *n* = 11) and were analyzed by nonparametric Mann-Whitney *t*-test. Error bars show the mean ± SEM. *** *p* < 0.001, **** *p* < 0.0001, ns not significant

We previously reported that MEKi reduced alveolar neutrophil inflammation after LPS-induced ALI [3]; however, a major concern regarding immune-modulating therapeutic strategies in live bacterial infections is the potential impaired pathogen clearance, especially if leukocyte inflammation is reduced below a critical concentration required for effective bactericidal activity [12]. To determine if MEKi altered lung clearance of a clinically relevant bacterial pathogen, mice were infected with 5 × 10^6^ CFU of *P. aeruginosa* in an experimental pneumonia model. Infection resulted in acute illness as indicated by weight loss (Fig. 1a) and reduced physical activity (observed but not quantified) in mice on days 1 and 2, post-infection. In a treatment strategy following the onset of infection, MEKi or carrier control was administered by i.p injection to groups of mice on days 2 and 3, post-infection. MEKi-treated mice had significant recovery of weight on day 3 compared to carrier-treated mice (Fig. 1a). On day 4 after infection, bacterial clearance from the lung was not altered between carrier and MEKi-treated mice (Fig. 1b), and importantly, MEKi did not result in dissemination of *P. aeruginosa* to the spleen (data not shown).

The study also measured the total number of cells in the BAL from mice on day 4 post-infection and found that MEKi-treated groups had reduced BAL cell counts compared to carrier-treated controls (Fig. 1c). Differential analyses revealed that MEKi significantly reduced neutrophils compared to carrier-treatment, but did not alter macrophage numbers (Fig. 1c), similar to findings during LPS ALI [3]. Uninfected groups treated with carrier control or MEKi demonstrated no effect of MEKi on weight (Fig. 1a), BAL cell counts (Fig. 1c), or BAL cell differential (data not shown). As additional characterization of MEKi-treatment, total protein and IgM levels in BALF were measured as indicators of lung injury and vascular leak. While the three uninfected groups were near the lower limits of detection for both total protein and IgM (Fig. 1d), infected mice had elevated levels of both total protein and IgM, although MEKi-treatment had no effect on these levels at this time point. In summary, MEKi delivery after infection was able to reduce some parameters of inflammation, such as neutrophil levels, and improve weight recovery without inhibiting bacterial clearance.

### MEKi alters macrophage responses in vivo during resolution of inflammation

**Fig. 2:**
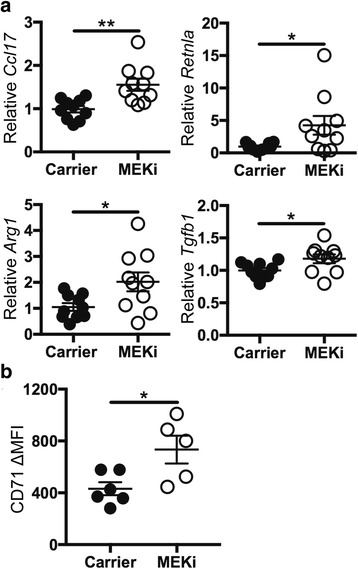
MEKi modulates in vivo macrophage polarization. Alveolar macrophages were obtained from BAL **a** and lungs **b** from C57BL/6J mice 4 days after infection with 5 × 10^6^ CFU *P. aeruginosa* that received carrier or MEKi treatments delivered by i.p. injection on days 2 and 3 after infection. **a** Alveolar macrophages from BAL were isolated and used for measurement of M2 gene expression. The levels of *Ccl17*, *Retnla*, *Arg1*, and *Tgfb1* were measured relative to *Hprt* control and data were normalized to carrier-treated animals; *n* = 10/group. Error bars show the mean ± SEM, analyses used parametric unpaired *t*-test. **b** Single cell suspensions of cells obtained after lung digestion were used for staining and flow cytometry analyses. Alveolar macrophages were identified as CD45^+^/Ly6G^−^/CD11c^+^/SigF^+^ cells and the ΔMFI of carrier (*n* = 6) and MEKi (*n* = 5) treated mice from one representative experiment of two are shown. Error bars show the mean ± SEM, analyses used parametric unpaired *t*-test. * *p* < 0.05, ** *p* < 0.01

During the resolution of lung injury, macrophage gene and protein expression changes from a pro-inflammatory M1 profile to a reparative M2 profile [11, 13–15]. It was observed that MEKi treatment in vitro and in vivo increased macrophage M2 gene and protein expression [3] and we hypothesized that MEKi would also modulate macrophage functions during the resolution of live bacterial *P. aeruginosa* pneumonia. To determine if MEKi enhanced macrophage M2 phenotypes, gene expression from alveolar macrophages obtained from the BAL of mice at day 4 post-infection was examined. Compared to carrier-treated mice, alveolar macrophages from MEKi-treated mice had increased expression of M2 genes *Ccl17*, *Retnla*, *Arg1*, and *Tgbf1* (Fig. 2a). Uninfected mice that received either carrier or MEKi did not have increased expression of these genes when compared to uninfected naïve mice (data not shown). In addition, the study examined surface expression of the transferrin receptor CD71, another M2 marker, on alveolar macrophages that were identified by flow cytometry as CD45^+^/Ly6G^−^/CD11c^+^/SigF^+^. Similar to the findings after LPS ALI, MEKi was found to increase alveolar macrophage surface expression of CD71, compared to carrier-treated mice (Fig. 2b). Collectively, these data support the notion that MEKi has immunomodulatory properties that alter macrophage programming to promote increased M2 gene and protein expression associated with increased resolution of lung injury [3].

## Discussion

*P. aeruginosa* is an opportunistic pathogen that can induce significant lung inflammation and tissue damage, for which new immune modulating therapeutic strategies are needed [16]. One concern of anti-inflammatory therapeutics is the potential for impairment of host–defense mechanisms involved in pathogen clearance, resulting in increased susceptibility to infection. This study examined the ability of MEKi to promote the resolution of inflammation during an experimental model of *P. aeruginosa* pneumonia. Importantly, the data indicate that MEKi did not impair clearance of pulmonary *P. aeruginosa* infection. No difference in lung CFUs was observed, and no bacterial dissemination to the spleen was detected 4 days after infection. MEKi treatment decreased alveolar neutrophilic inflammation and led to faster recovery of weight, which we interpreted as beneficial effects. Given reduced BAL neutrophils in infected MEKi-treated mice, our study looked at whether MEKi treatment reduced the level of the neutrophil chemokine, CXCL1/KC, in the alveolar compartment. The levels of CXCL1/KC in the BALF at day 4 in uninfected and infected mice treated with MEKi or carrier control were measured. At this time point, CXCL1/KC were near the lower limit of detection for this assay and were approximately 100-fold lower at this time point compared to what has previously been observed within 4 h of *P. aeruginosa* infection [11]. However, despite these low levels, our study did not find a decrease in CXCL1/KC levels in MEKi-treated mice compared to carrier-control mice to explain reduced BAL neutrophil numbers (not shown). In addition, although MEKi did not alter BAL total protein or IgM levels at the time point examined, it is unknown if MEKi reduces these indicators of lung injury at earlier times after treatment. For example, the levels of BAL IgM peak within the first 24 h after infection and then decline [14, 17]. Hence, it is possible that delivery of MEKi may need to be initiated earlier to effect a change in markers of vascular leak or to detect differences in CXCL1/KC.

In summary from the data presented here, MEKi-treatment appears to modulate some, but not all, indicators of ALI. Macrophage M2 programming is increased during the resolution of ALI and it has recently been identified that MEKi increases macrophage M2 polarization in vitro and in vivo [3, 13]. Here, further data that support this notion is presented, showing that MEKi increased several markers of alveolar macrophage M2 polarization after *P. aeruginosa* infection, which may contribute to, or be a marker of, more rapid resolution of lung inflammation in MEKi-treated mice. These results contribute additional evidence that MEK1/2 inhibition has the potential to dampen pro-inflammatory responses and increase anti-inflammatory responses in murine models of infection and inflammation [3, 5, 6, 9].

As with all potential anti-inflammatory agents, there are concerns about increasing susceptibility to infection due to impaired host-defense mechanisms. Several experimental studies have evaluated the use of MEKi-treatment in the rat and mouse cecal-ligation and puncture (CLP) models of sepsis, demonstrating therapeutic anti-inflammatory effects without altering bacterial burdens [5, 8]. However, these models are limited in their scope of evaluation for several reasons. First, utilizing a pre-treatment regimen does not fully capture the clinical utility of MEKi as an anti-inflammatory in patients who present with inflammatory lung disease/infection. Second, the limited duration of time (< 24 h) and anatomical location of the CLP model does not allow for evaluation of a primary lung infection and potential alterations in lung host–defense mechanisms. A study examining both pre- and post-infection treatment with a MEKi during primary lung infection was recently reported [18]. However, this study utilized a lethal *F. tularensis* murine infection model, which results in rapid and extensive dissemination of infection from lung to the liver and spleen [18], therefore not allowing examination of whether MEKi has detrimental effects on pulmonary host defense mechanisms.

Although prophylactic pre-treatment drug strategies are utilized in many pre-clinical studies to maximize potential beneficial results, therapeutic strategies employed post-infection are most likely to simulate clinical applications of a potential drug, which is a strength of this study design. Under this premise, these data indicate that MEKi is capable of promoting the resolution of inflammation and increasing macrophage M2 polarization, similar to what was previously observed during experimental LPS-induced lung injury [3]. Additionally, recent findings have demonstrated that MEK1/2 inhibitor treatment can dampen the inflammatory response and provide a beneficial outcome when applied in a mouse model of cerebral malaria, in part by increasing IgM production by B1 cells and increasing the phagocytic activity of macrophages and neutrophils [9].

There are several limitations to the current study. First, based on published studies that demonstrate peak injury and inflammation occurring at days 2–3 after infection [11, 14, 17], we chose to model the effect of MEKi during this period with assessment of inflammation on day 4 after infection. However, it is recognized that examination of MEKi-treatment at both earlier and later time points are necessary to gain a more meaningful understanding of MEKi effects on inflammation and resolution. A primary goal of the current study was to determine if MEKi-treatment acutely impaired the ability of mice to control infection; however, whether MEKi alters susceptibility to recurrent or polymicrobial infections remains to be examined. Future studies will examine the mechanism by which MEKi reduces neutrophil levels in the lung. While the current data did not support a role for reduced CXCL1/KC at day 4, it is possible that CXCL1/KC, or additional neutrophil chemokines, may be altered by MEKi at earlier time points after treatment. Alternatively, previous studies found that MEKi enhances expression of receptors involved in efferocytosis and increases clearance of apoptotic polymorphonuclear cells (PMNs). Whether MEKi enhances macrophage efferocytosis in the lung or alters intrinsic properties of neutrophil migration should be investigated [3, 9]. While all of the effects of MEKi treatment are not fully understood, this study demonstrates that MEKi delivery did not impair bacterial clearance during acute infection. It is anticipated that further investigations into the use of MEKi as an anti-inflammatory therapeutic during recurrent or chronic *P. aeruginosa* infection models will yield additional insights into the potential beneficial effects and mechanisms of immune modulation.
